# Acknowledgment to the Reviewers of *Bioengineering* in 2022

**DOI:** 10.3390/bioengineering10020134

**Published:** 2023-01-19

**Authors:** Bioengineering Editorial Office

**Affiliations:** MDPI AG, St. Alban-Anlage 66, 4052 Basel, Switzerland

High-quality academic publishing is built on rigorous peer review. *Bioengineering* was able to uphold its high standards for published papers due to the outstanding efforts of our reviewers. Thanks to the efforts of our reviewers in 2022, the median time to first decision was 15 days and the median time to publication was 40 days. Regardless of whether the articles they examined were ultimately published, the editors would like to express their appreciation and thank the following reviewers for the time and dedication.
Abbas RahdarLouis Jun Ye OngAbdelfatah KouidereLouis Wy LiuAbdelhameed IbrahimLuan Luong ChuAbdollah AllahverdiLuca CavaggioniAbdou HachaniLuca FortiAbdul K. ParchurLuca MenichettiAbdulrahman AlwarthanLuca MesinAbhay K. PandeyLuca MoscettiAbhishek KandwalLuca RegusciAbideen ZainualLuca Salvatore De SantoAbraham PouliakisLucia MacchiusiAdam C. PalmerLucia MorbidelliAdam E. GawedaLuciana Biagini LopesAdam MiranowiczLuciana Porto De Souza VandenbergheAdam StroińskiLucie HasoňováAdão SilvaLuguang WangAdawiya J. HaiderLuigi FortunaAdil AkkouchLuigi LoscoAdina NegreaLuigi ScipioneAditi ChopraLuis Adrián Zúñiga AvilésAdrian HarrisonLuis Alberto Morales RosalesAdrián LozanoLuis CoelhoAdrian NeaguLuís Giner-TarridaAdriana Gadioli TaroneLuis RodrigoAdriana TrifanLuísa SerafimAfaque Manzoor SoomroLuiz Palucci-VieiraAfsheen TabassumLukasz JaremkoAfzal HussainLukasz KozlowskiAgata StanekLukasz MalekAgnès DrochonLuminita AndronicAgnes KlarLuminita MoraruAgnese SbrolliniLung-Kwag PanAgnieszka KrakowiakLu-Ting KuoAgnieszka Mazur-BialyLyudmila DimitrovaAgnieszka NowakM. Akhil. JabbarAgnieszka TorzewskaM. G. SumithraAgnieszka WareńczakM. R. MozafariAgnieszka ZagórskaMa Del Rocio López-CuellarAgustin L. Herrera-MayMaarouf AdilAhmad SaltiMaciej SkotakAhmed AbassMaciej ŻukowskiAhmed Abdal DayemMadhur UpadhyayAhmed E. KholifMafalda LaranjoAike QiaoMagdalena MatczukAisha FarhanaMagdalena Zabochnicka-ŚwiątekAjay Kumar ChinnamMagdalena ŻychowskaAjay Kumar YagatiMagdalene J. SeilerAjay PalMahendran SekarAjil JalalMahmood AhmedAjinkya PawarMahmood BaraniAkash Kumar BhoiMahmoud AbulmeatyAkihiko HiyamaMahmoud DiabAkihiko YamamotoMahmoud ElbattahAkira MonjiMahmoud ElsisiAkira ObanaMahmoud WagihAkishige HokugoMahmoudreza HadjighassemAkshay ModiMailin GanAlain Favre-RéguillonMajid VahedAlan HarperMajumder SubrataAlbert E. PattersonMakoto EndoAlbert FujakMakoto TsunodaAlbert PoaterMakusu TsutsuiAlberto AmarettiMalek Ben Ahmed AdouniAlberto Dominguez VicentMalgorzata LobockaAlberto PozzoliMałgorzata ŚwiętekAlberto Rodríguez-ArchillaMałgorzata WrzosekAlbino EccherMan-Fai LeungAlejandro Puente CastroMangesh HadeAleksandr BulaevManlio SantilliAleksandra KrólickaManoj Kumar TembhreAleksandra Piechota-PolanczykManuel DoblaréAleksandra ZgrundoManuel LagacheAleksey A. UstyugovManuel Marques FerreiraAles StrancarManuel MataAlessandra LuccheseManuel Nieto-DiazAlessandra PiscitelliManuel Prado-VelascoAlessandro De SireMan-Yeon ChoiAlessandro OrtisMar Llamas-VelascoAlessandro PoggiMarcel HeersAlessandro RizzoMarcin ArciszewskiAlessandro VolonterioMarcin DębowskiAlessio GizziMarcin SosnowskiAlex KuchumovMarcin SylwestrzakAlexander BerezinMarco CaricatoAlexander E. KabakovMarco CascellaAlexander H. MaassMarco CicconeAlexander HawlitschkaMarco DolciAlexander KamnevMarco GovoniAlexander M. KorsunskyMarco GrossiAlexander S. NovikovMarco NaiaAlexander ShmakovMarco NisiAlexander VasilyevMarco PortelliAlexander Yu MeigalMarco RoccettiAlexandra TǎbǎranMarco SeracchianiAlexandru Vasile RusuMarcos Rodríguez MillánAlexei SolovchenkoMarcos ZeddaAlexey G. KolmakovMarcus D. LanceAlexey NikulinMarek CzosnykaAlfonso IbarraMarek K. DobkeAlfonso Maria PonsiglioneMarek KozińskiAlfonso MastropietroMarek MuriasAlfonso Olaya AbrilMarek WojnickiAlfredo IandoloMaria Angeles RojoAlfredo Téllez-ValenciaMaria Angelica MiglinoAli Akbar AshkarranMaria Carla CraveroAli ZarrabiMaria Conceição MansoAlice BerardoMaria Cristina De ColaAlice BonomiMaria D. Rikkou-KalourkotiAlice PievaniMaria Giovanna CilibertiAlice Verticchio VercellinMaria Giovanna FrancipaneAlireza AhmadianMaria Helena FernandesAlireza KarimiMaria Helena Melo LimaAlmir BadnjevicMaria I. PiloÁlvaro Gabriel Sepúlveda MartínezMaria Isabel Achaerandio PuenteÁlvaro MeanaMaria Isabel Ribeiro DiasAlvaro YogiMaria João PeixotoAmandine MagnaudeixMaria KoufakiAmeeduzzafar ZafarMaria RizziAmeersing LuximonMaria Serena ChiriacòAmin HekmatmaneshMaría Vivero-LopezAmin R. JaverMaria ZanklAmir Hossein OveisMarian SimkaAmir WaseemMariana FloriaAmit BansalMariano Francesco CaratozzoloAmit JaiswalMariano García-ArranzAmjad Jaleel HumaidiMarijana PetkovićAmjad RehmanMarilena GrignanoAmmad Ahmad FarooqiMarilena PannoneAmmar ArmghanMarina GallarateAmrit KaphleMarina PiscopoAmrita ChawlaMarina QuartuAmro AmaraMario A. PagnottaAna FonsecaMario Bernardo-FilhoAna Paula PiedadeMario D’AcuntoAna Rita Costa-PintoMario DiazAnahita Jablonski-MomeniMario MeroneAnand ThirupathiMario MolinaraAnantha HarijithMário S. RodriguesAnastasia TsingotjidouMario SabatelliAnca DinischiotuMario ValentinoAndjela DraganićMário VéstiasAndjelka KovačevićMariusz MojzychAndrás KincsesMark BegoniaAndre LopesMark ClemensAndre Luiz CostaMark F. PittengerAndré MérmoudMark P. HeitzAndre RodackiMark PostAndrea ButeraMark S. JohnsonAndrea CochisMark ScherAndrea EhrmannMark Ter LaanAndrea PatriarcaMarkku PenttonenAndrea RuffiniMarko SamardžijaAndrea SambriMarkus A. WimmerAndrea SistiMarkus RuetzAndrea SummerMarta Dyszkiewicz-KonwińskaAndrea TigriniMarta KepinskaAndrea VasconsueloMarta KlakAndreas ManiosMarta KopaczyńskaAndreas RiesMarta S. CarvalhoAndreea IacobMartin DegenAndrés Amador Garcia-GranadaMartin KollerAndres Barajas-SolanoMaryam Mobed-MiremadiAndrew RuysMaša Knez MarevciAndrew WestwellMasafumi MurataniAndrey A. KovalevMasahiro ImamuraAndrey KrauklisMasamichi KamihiraAndrzej MaterkaMasamitsu HyodoAndrzej PolańczykMasamitsu KanadaAndrzej ŚliwczyńskiMasao KodaAndrzej SlominskiMasaomi IkedaAndrzej Za̧BekMasashi ToyodaAndy Wai Kan YeungMasato YasumotoAneta A. PtaszyńskaMasayuki TsunekiAneta SkaradzińskaMasood KhanAng-Chen TsaiMasoumeh Rajabi BazlÁngel Serrano-ArocaMasrina Binti Mohd NadzirAngela NebbiosoMassimiliano De PaolisAngela Rubio-MoragaMassimo CapocciaAngela SorrentinoMassimo GalliAngia Sriram Pradeep RamMassimo MaccagnoAnil SinghMassimo MoroAnita ŠalićMassinissa YahiaAnn C. FoleyMatej KranjcAnna JelińskaMateja J. PrimožičAnna Kasielska-TrojanMatteo BolcatoAnna MichnikMatteo SaccucciAnna MichopoulouMatthew PediaditisAnna Rafaela Cavalcante BragaMatthias GüntherAnna Szafranek-NakoniecznaMaurizio MuracaAnna TrubetskayaMaurizio PascadopoliAnne Mari RokstadMauro D'ArcoAnne VanetMauro MalvèAnne-Lyse DucrestMax MenziesAnne-Sophie WoznyMaxime TourteAnton BonartsevMd Jamal UddinAnton KiselevMd. Abdul AwalAnton KutikhinMehdi RazaviAnton MoritzMehedi MasudAnton V. NaumovMehmet GülAntonella LisiMehmet Hamit OzyaziciogluAntonella MattanaMehrdad RostamiAntonello SantiniMejdi SnoussiAntonietta MessinaMekala VenkatachalamAntonio CicchellaMekdimu Mezemir DamtieAntonio CorteseMelanie Haffner-LuntzerAntonio D. MorenoMengqi ZhangAntonio GloriaMeysam TayebiAntonio RamosMichael Bruce JarstferAntonio SteccoMichael D. DiamantidisAntonio TrovatoMichael DöllingerAntonis SakellariosMichael EckerstorferAntony KamMichael EichbaumAnuj KumarMichael G. WellerAnup S. PathaniaMichael J. McclureAnuraag BoddupalliMichael MangoldAnwar UsmanMichael OlsonApostolos D. GalatosMichael RaghunathAqeel AhmadMichael SacksAranzazu VillasanteMichał GinsztArcady PutilovMichal JurášekArchana SinghMichał LatalskiAriana GattMichał StrzeleckiAriel Colaço De OliveiraMichela BosettiArif Md. Rashedul KabirMichela FerrucciArindam PaulMichele BoffanoArkadiusz JózefczakMichele MaffiaArkadiusz OrchelMichele MalgeriArkady SerikovMichelle OyenArkady VoloshinMieszko WieckiewiczArley D. ZapataMiguel A OrtegaArmin RajabiMiguel AlcarazArnaud BertschMiguel LaderoÁrpád Ferenc KovácsMiguel Ponce-VargasÁrpád TósakiMihaela DinuArputharaj Joseph NathanaelMihaela HnatiucArrigoni FedericaMihai CenariuArtem BelesovMiho NakamuraArthur De Sá FerreiraMikhail AvdeevArtur Tadeusz KrzyżakMikko KanervaArtur TaszakowskiMiklós KubinyiArturo Cadena RamírezMilan GnjatovićArturo Forner-CorderoMilan KubiatkoArun ButreddyMilan MilivojevićArunkumar RengarajMilica PešićArutselvan NatarajanMiljan MilosevicArvind Kumar ShuklaMina BaniasadAshfaq AhmedMinaxi SharmaAshish B. DeoghareMindra Eugenia BadeaAshish JainMinfang ZhangAshkan BighamMing LiuAshu JohriMingchong YangAsif KhanMing-Fong TsaiAtefeh ZarepourMingfu ChenAthanasios PapakyriakouMinghua HoAtsuyuki InuiMing-Kuei ShihAttila BendeMingyue DingAttila TordaiMingze SunAura RusuMingzhe JiangAvgis HadjipapasMinhao XieAyataka FujimotoMiomir KnezevicAydin Bordbar-KhiabaniMirja AhmmedAyman ElbazMiroslav BačaAyman MostafaMiroslaw JanowskiAzadeh AsefnejadMirta MilićAzzurra StefanucciMititelu Tartau LilianaBabak ChehroudiMitsuoki KawanoBabasaheb MatsagarMiusi ShiBahrad A. SokhansanjMizuki TanakaBalaji PanchapakesanMohamed Abd ElazizBaltatu SimonaMohamed Abdullah JaberBarbara GhinassiMohamed Adel HammadBárbara Muñoz PalazonMohamed SassiBarbara PavanMohamed SharafeldinBart PijlsMohamed TrabiaBasilio PueoMohammad Adel GhiassBasilio VescioMohammad AlkilzyBeat GöpfertMohammad AshfaqBeata KuczyńskaMohammad Bagher DowlatshahiBeatriz Xoconostle-CázaresMohammad JamshidiBen GreenebaumMohammad Javed AnsariBenedikt Johannes BraunMohammad Khursheed AlamBernard SpeculandMohammad MobashirBernardo InnocentiMohammad Nazmul HasanBernat SoriaMohammed Al-SaremBernd W. SiguschMohammed DiykhBert VandenberkMohammed F. HamzaBertrand LiagreMohammed Rafi ShaikBessem MkaouerMohan KaruppayilBibha ChoudharyMohd Asyraf ZulkifleyBimal GhimireMohd Ikram AnsariBin ChenMohd KhanapiBin ZhengMohd Normani ZakariaBingpu ZhouMohd TanveerBinoy MaitiMohsen YazdanianBiserka RelicMojtaba AnsariBishnubrata PatraMokarram HossainBishweshwar PantMominur RahmanBo ZhengMona I. ShaabanBogdan GhermanMonika CzerwińskaBogdan SoceaMonika KijewskaBogdan TiguMontserrat GarcíaBohan YinMontserrat TobajasBoris B. StraumalMorgane A. EvinBoris KozlovMoritake IguchiBoris U. StambukMosab KaseemBoros MihályMoses OyewumiBor-Show TzangMostafa KhattabBrahim BchirMoustafa R. K. AliBrett HamblyMuhammad A. RahmanBrian BothnerMuhammad Adnan AsgharBrian D. AdamsMuhammad Azam KhanBrian HelenbrookMuhammad Iqbal R. KhanBrian M. WardMuhammad Mubasher SaleemBrigitte AltmannMuhammad N ZahidBrij GuptaMuhammad NadeemBrijesh SinghMuhammad NawazBruce MilthorpeMuhammad NazirBruce T. VolpeMuhammad Nidzhom Zainol AbidinBruno AndréMuhammad RasoolBruno ChrcanovicMuhammad Shahid Riaz RajokaBruno Nicolau PaulinoMuhammad Sohail ZafarBryan FullerMuhammad ZamanBulat AkhmadeevMuhsin J. JweejByungheon LeeMullaicharam BhupathyraajByung-Hoon KimMun'Delanji C. VestergaardC. K. SumeshMurali DamaCalin VaidaMurugabaskar BalanCamilla LuniMurugesan VelayuthamCamille DuranMushriq AbidCamilo Zamora-LedezmaMustafa BecerikliCarl ErbMustapha HidouriCarlo AlfieriMuzamil Majid KhanCarlo CosentinoNada SallamCarlo DrioliNadeem AbbasCarlo LajoloNadim ZgheibCarlo RicciardiNadine NagyCarlos CaroNagappan KrishnarajCarlos CaulinNagavendra KommineniCarlos Cuevas-SuárezNaji KharoufCarlos Duarte-GalvanNalladurai KaliyanCarlos E. Galván-TejadaNan ZhaoCarlos Eduardo BaccinNanasaheb.D ThoratCarlos Eduardo Molina-GuerreroNanditha AnandakrishnanCarlos EscobedoNaoki YamamotoCarlos EstrelaNaoya KakimotoCarlos Fernando MourãoNapoleão Martins Argôlo NetoCarlos Miguel MartoNarendra Pal Singh ChauhanCarlos Roberto GrandiniNark-Kyoung RhoCarlos Roberto Hall BarbosaNarongchai AutsavaprompornCarlotta GranchiNarongrit MuangmaiCarmelo MilitelloNashwa HagagyCarmen C. LlenaNasim ChiniforushCarmen Moret-TatayNasim Nsooudi NsooudiCarolyn M. PrimusNatalia GudzCatalin StoeanNataliya V. StoynovaCatalina Natalia Cheaburu YilmazNatalya KizilovaCaterina LiciniNatasa ZarovniCelina Maria DamianNathan TannerCesar OzunaNatheer KhasawnehCesare D’AmicoNatividad Gómez-CerezoCesare ZoiaNattapol AunsriChamila GunathilakeNavapon TechakriengkraiChamutal BornsteinNazia HameedChandrakala Aluganti NarasimhuluNeelamadhab PadhyChang XiaojunNehal ElsherbinyChao MiNermeen Adel ElkasabgyCharalampos SiristatidisNeusa MartinsCharles B. HuddlestonNewcomb RobertCharles D. EggletonNguyen Quoc Khanh LeChen Huei LeoNhien Thi NguyenChen ZhuNiall BarronChengyi TuNibedita LenkaChenhan ZhongNicholas J. KenyonChen-Hua LiuNicholas VlachosChenxi YangNickolay N. SmirnovCherie KrugerNicola CirilloChe-Yu LinNicola MarottaChi Bum AhnNicola MontemurroChi LeeNicolae GicaChia-Che HoNicoletta ZermanChia-Hua LiangNicos MaglaverasChian Ju JongNidal JaradatChian-Hui LaiNihan AydemirChiara Giulia FontanellaNikhil KhannaChiara Maria MottaNikola AnđelićChien-Ming ChaoNikolaos KourkoumelisChih Jer LinNima MovahediChikashi SatoNing LiChin-Chean WongNipon Theera-UmponChing-Chi HsuNitin CharbeChing-Wei WangNivaldo Antonio ParizottoChinmaya MahapatraNobuto NakanishiChiranji Lal ChowdharyNobuyuki IshibashiChiwan KooNoha H. HabashyChoon Weng LeeNomso Hintsho-MbitaChris H. L. LimNongyue HeChris JonesNorbert ZolekChristian W. OmlinNorihiko KamiokaChristine Müller-RennoNorihiro SuginoChristoph BuchtaNorman R. WilliamsChristopher EhrhardtNorman Scott LitofskyChristopher KobierzyckiNuno M. GarciaChrysoula VoidarouNur MustafaogluChuanlong GuoNuri AzbarChuanzhen ZhaoNurul Asma AbdullahChuda ChittasuphoO. Roger AndersonChun Hung ChuObireddy Sreekanth ReddyChung How KauObyedul Kalam AzadChung-Ming ChenOctavian PostolacheChung-Sung LeeOleg V. BatishchevChunmei CaoOlga KopachChunyi WuOlga TrhlíkováCid Marcos Gonçalves AndradeOlga ZeniCindy H. ChauOlivier LaprévôteCinzia GiacomettiOmneya AttallahCinzia MasperoOriana TrubianiCiro EspositoOsamu IshidaClare YellowleyOscar Ruiz-SalgueroClaudia DettoriOsvaldo MazzaClaudia MatteucciOthon PapadopoulosClaudio NarduzziOveis PourmehranClaudio ZuccaPablo BoixedaConcetta Di NoraPablo G. Del RíoCongxu ZhuPamela A. MoalliConstantino Carlos Reyes-AldasoroPanagiota KatikouConstantinos LoukasPanagiotis PeitsidisConstantinos ZervidesPankaj KumarCornelia KasperPaola De PadovaCostantino BalestraPaola Sanjuan-AlberteCostantino Del GaudioPaola SavoiaCostin Teodor StrebaPaolo BennaCrescenzio GalloPaolo CapparèCristian SimionPaolo DapavoCristian TrâmbițașPaolo FagoneCristian Tudor MateaPaolo FuschiCristiane Koga-ItoPaolo ViscontiCristina BignardiPaolo ZaffinoCristina Manuela DrăgoiPapović SnežanaCristina RicartParaskevi MitliagkaCristina SatrianoParracino AntoniettaCristina SoaresParthasarathi SubramanianCsaba HegedűsParvaneh SamanCüneyt M. AlperPasquale MarrazzoCurtise Kin Cheung NgPasquale PisapiaCyrille BlondetPaul AbbyadCyrus MotamedPaul FedakDa SunPaul HavigDag ØrstavikPaul SchoenhagenDag Roar HjelmePaulina KoczurkiewiczDalia ElsheakhPaulina RusanowskaDamien ChablatPaulo J. PalmaDan VârbanPaulo SantiagoDan WuPaulo ValeDana CopotPaulo Victor Sgobbi De SouzaDana TolomanPaulo ZainiDandan SunPavel A. ShilyaginDang Ngoc Hoang ThanhPavel KhramtsovDaniel C. NelsonPavel MakarevichDaniel DawsonPavel StarovoitovDaniel García IglesiasPavol BožekDaniel GilletPawan KumarDaniel H. FarkasPaweł ChmielarzDaniel KronenbergPaweł GaćDaniel MierlitaPaweł LinekDaniel S. OhPedram AzariDaniel ZakowieckiPedro FernandesDaniel ZwahlenPedro FonteDaniela BuchaimPedro MunueraDaniela Di VenerePeichao ChenDaniela MiricescuPei-Gen RenDaniele GiansantiPeihua MaDanish JaveedPeng ChenDanka BukvickiPengchao KangDante Mújica VargasPengfei WangDanuta JaworskaPentti NieminenDarin ZertiPeter GuttmannDario ColettiPeter HowellDariusz MikołajewskiPeter IssingDariusz T. MlynarczykPeter Physick-SheardDavid CaballeroPéter PótiDavid DolivoPeter WinloveDavid E. AndersonPeter ZiouposDavid HaynesPetra KoraćDavid L. CarawayPhilip J. SteerDavid LeavesleyPhilippe BillialdDavid LevinPhilippe SoyerDavid McgiffinPia Lopez-JornetDavid Neil MannersPierluigi CarcagniDavid O'SullivanPiero Antonio ZeccaDavid PerpetuiniPiero FranceschiDavid PoynerPiero PaladiniDavid SanterPierre BongrandDavood KharaghaniPierre NassoyDavood RahmatabadiPierre-Simon JoukDawid PolapPietro ScicchitanoDawid StefaniukPinaki BhattacharyaDawid SzwedowskiPinelopi SamaraDaylon JamesPiotr KaczmarekDayun YanPiotr LulińskiDeane E. SmithPiotr PiszczekDebabrata MandalPiotr ProchorDeepak Kumar KhajuriaPiotr Wa̧ŻDemetrios ArvanitisPiyush BaindaraDeng WangPo-Lei LeeDeng-Guang YuPoonam SharmaDenis SilachevPo-Ting WuDennis FlanaganPrabhakar OrsuDerek RosenzweigPrabhakar SharmaDerya BirantPradeep KumarDésirée WünschPradeep Kumar SinghDesmond AgboadaPradipta Ranjan RautaDhiraj BhatiaPrasanna NeelakantanDi ZhangPrashanth RavishankarDiana LousaPrince ChawlaDianbao ZhangPriti Prasanna MaityDiego MinciacchiPunita K. LalDigamber RaneQaiser RiazDiming ZhangQamar AbbasDimitrios BikiarisQi GuDimitris DrikakisQi ShaoDimitris ZavrasQi XiangDinesh Veeran PonnuveluQiang LaiDingwen ZhangQiang LiuDirk KucklingQiang ZhuDolores R. SerranoQichang MeiDomenica CapassoQiguo RongDomenico CiavarellaQijun HuangDonatella Degl'InnocentiQin FuDong ChenQing ZhangDong-Woo ChoQing ZhouDong-Wook HanQingwei LiaoDonna WallQize ZhangDorianna SandonàRabha W. IbrahimDorin GheorgheRabiu MusaDorina StriethRachel A. OldershawDorota PuchowiczRachel LiDörte SolleRade M. BabićDragana Ž. KrstićRadek MalikDrago DolinarRadomir JasinskiDuarte MarquesRadosław GocołDuc Dung NguyenRafael Fernández-MartínDuo Wai-Chi WongRafael LlobetDurgarao GijjapuRafael Salto-GonzalezDusan MiticRafał BielasDwaipayan MukherjeeRafał WienchDzati Athiar Bt RamliRaffaele De FrancescoEbrahim GhaderpourRaffaele SerraEdgar López-LópezRaffaella BucciEduard GuaschRaghavan Pillai RajuEduardo Goncalves MotaRaghvendra GuptaEdwin-Joffrey CourtialRahmat EllahiEfstratios I. KamitsosRaj BhullarEhsan AminRajesh Kumar TripathyEijiro AkasakaRajkumar Kottayasamy SeenivasagamEiriki SunamuraRajni VermaEitoyo KokubuRaksawan DeenonpoeEkaterina BaryshnikovaRalf TakorsEkaterina NaumenkoRaluca IanchisElaine KaspchakRaluca NicuEleana KontonasakiRamar ThangamEléanor LuceRamesa Shafi BhatEleftherios TouloupakisRamesh NagarajappaElena CaporaliRamgopal KashyapElena DilonardoRamiro VelázquezElena DonettiRamón Alberto Mollineda CárdenasElena JonesRamy Abou GhaydaElena Martinez-SanzRamya Lakshmi RajendranElena SvirshchevskayaRamzi MahmoudiEleuterio A. Sánchez-RomeroRanga Rao AmbatiElham AhmadianRanjith PremasiriElie MassaadRaoul ReiserEliete CaldeiraRaquel Leirós-RodríguezElisa BelluzziRaquel Moreno LoshuertosElisa CairrãoRaquel Sánchez-RodríguezElisabetta CanettaRasoul MirzaeiElisabetta PolizziRattana JariyaboonElizabeth Carvajal-MillanRaul ArrabalElizabeth VinodRaul DomínguezEllen GoldmanRazieh RazaviElquio Eleamen OliveiraRebai Ben AmmarElsa C. ChanRedha TaiarEly ValbuenaRegina Maria Puppin-RontaniEmanuela ChiarellaRégulo López-CallejasEmanuele CarnielReinaldo Rodríguez RamosEmiliano LaudadioRenato GalzioEmma C. CacciolaRenato TorresEmmanouil KapetanakisRené M. CasteleinEmmanouil PapanastasiouRenzo CorderaEmre KarakusRenzo GuarnieriEnrico GherloneRiadh BadraouiEnrico GiordanRicardo Faria RibeiroEnrico RagniRicardo Hernández-MartínezEnrique BerjanoRicardo VardascaEnrique Gomez GomezRiccardo BeltramiErfan MalekiRiccardo LombardoEric HuetRichard G. HaverkampEric SniderRino RappuoliErin H. SeeleyRita ChiaramonteErshuai ZhangRizwan QureshiErsilia NigroRobert A. HorowitzEsam Bashir YahyaRobert C. FowkesEsmaeel SharifiRobert DickinsonEssam Al-MoraissiRobert MusiołEstanislao Nistal-VillánRobert RekawieckiEszter DuczaRobert SifrerEtsuko KobayashiRobert Steven EsworthyEugene C. LinRobertas DamaševičiusEugene SkourasRoberto de FazioEugenia BezirtzoglouRoberto GramignoliEugenio ProvenzanoRoberto GristinaEui Soo KimRoberto Li VotiEulicio De Oliveira Lobo-JuniorRoberto Lo GiudiceEva M. Santos LópezRoberto Sanchis-SanchisEvangelia VouvoudiRoberto TeghilEvgenia SitnikovaRobin A. FelderEvgeniya BurkovaRodica Maricela AnghelEwa JakubczykRodolfo PicchioEwa KorzeniewskaRodrigo A. Guzmán-VenegasEwald SchnugRoger FrétyEwelina CichońRoger J. GuilloryEwelina Świa̧Tek-NajwerRogério Leone BuchaimFabian ZanellaRohollah NasiriFabiana LucàRoksana MarkiewiczFabio Camacho-AlonsoRomany MansourFábio FernandesRomeo Petru DobrinFabio GrizziRonald B. MooreFabio Kurt SchneiderRongkung TsaiFabio ZobiRoopa BiswasFabrizio MontecuccoRosa CibriánFadis F. MurzakhanovRosa VallettaFahmi ZaïriRosario SarabiaFanette ChassagneRoseline Oluwaseun OgundokunFany ReffuveilleRosella CataldoFarrukh ChishtieRoss LeightonFarshid SefatRouhollah FathiFatemeh GhorbaniRóża DzierżakFatemeh YazdianRubén Alvarez-AlvarezFatih BuyuksonmezRubén González CrespoFatih HatipogluRuben San-SegundoFatih KarpatRuchi SharmaFawzia Bardag-GorceRüdiger MaschkeFederica BoschettiRudolf KieferFederico CerroneRui LiFei HeRui MinFei XingRui Pedro LopesFeipeng YangRuiguo YangFelice LorussoRumiana TzonevaFeng LiRushendhiran KesavanFeng QiRuud G. J. MeulenbroekFengjie SunRuud P. M. DingsFeng-Lin YenRuxandra Maria Ilie-MihaiFernanda GentilRyan M. HillFernanda Viana CabralRyoichi AsakaFernando Blaya HaroRytis MaskeliūnasFernando De La PortillaS. RamkumarFernando J. Aguilar-AyalaS. RioualFernando Vargas JuniorS. SmysFilippo MiglioriniSaad AlghamdiFiorella GuadagniSabine Kuchler-BoppFiorenzo MarinelliSabino PachecoFiras KobeissySabrina ConociFlavio BocciaSabrina MorelliFlemming AndersenSadiq HussainFlora N. BalievaSaeed M. QaisarFlorian Ion Tiberiu PetrescuSaeid KargozarFlorian WurmSahar MollazadehFlorin MiculescuSaif ur RehmanFlorina M. BojinSaikat Kumar BasuFlorina TeodorescuSajjad ShiraziFokko Pieter WieringaSakineh HajebrahimiFokko WieringaSakorn MekruksavanichFrancesc Miro MurSalvatore PetroneFrancesc Xavier AvilésSalvatore ValianteFrancesca AielloSamantha GinnFrancesca DiomedeSameer AwadFrancesco AgostiniSamer HaidarFrancesco AmentaSamet ÖzdemirFrancesco BiancoSami ObaidFrancesco BrandaSamira MalekmohammadiFrancesco CacciolaSamo FokterFrancesco Di LorenzoSampath Dakshina Murthy AchantaFrancesco NicotraSamuel Peña-LlopisFrancesco SpinelliSandeep Kumar MishraFrancesco StiloSándor BeszédesFrancesco TornabeneSandra DonniniFrancisca García-Moreno NisaSandra LindstedtFrancisco AlonsoSandra RodriguesFrancisco Guillen-GrimaSandro Da Silva CamargoFrancisco Manuel BaenaSangbum ParkFranco CervellatiSangho RohFrank E. GoodwinSanja StojanovićFrank KrauseSanjiban RoyFrank Vinholt SchiødtSanjoy GhoraiFrank WeberSanju GuptaFrans A. Van NieuwenhovenSanthosh ArulFranz L. DickertSantiago Gomez SalvadorFrederick SchoenSantiago J. BallazFredy KurniawanSantosh Kr. KarnFrieder W. SchellerSantosh KumarFucang JiaSaowarath JantaroFuyi WangSapana JadounFu-Yin HsuSara B. PereiraGabriel HancuSara CampinotiGabriel PiresSara FerrarisGabriela DudekSara I. AbdelsalamGabriela RâpeanuSara KhamsehGabriele CaviglioliSara ShimoniGabriele CervinoSara T. A. Al-RashoodGabriele DonatiSarah Brem SunshineGael Y. RochefortSarah PixleyGaetano PaoloneSaravanan MuthupandianGalliera Emanuela RitaSariya MapoungGanesh Dattatraya SarataleSaroj KumarGang LiSasa RajsicGary HooperSatish RainaGaskon IbarretxeSatoshi YamaguchiGaspare PalaiaSatoshi YokoseGavin WinstonSaturnino Marco LupiGebremariam BirhanuSatyendra Kumar MishraGenki YoshikawaSaulius BaleviciusGennaro VessioSavneet KaurGeorge BlanckSayan GangulyGeorge FragoulisSean Chun Chang ChenGeorge H. ThompsonSebastian BürkleinGeorge J. DiasSebastian ZahnreichGeorge KoliakosSeigo NishidaGeorge SamanidisSekar VijayakumarGeorgios GiarmatzisSelene BaroneGesa BeckSelim BozkurtGesmi MilcovichSengul DoganGhasem DiniSenka MeštrovićGheorghe LazaroiuSeong Soo A. AnGheorghe NechiforSeongjae JangGholam Hossein RoshaniSepanta HosseinpourGholamreza DaryaborSerafim M. OliveiraGhulam AbidSergey GudkovGhulam MuhammadSergio CaputiGiacomo DerchiSergio CasellaGiada GiorgiSeth PincusGiada MagniSever-Gabriel RaczGian Andrea PelliccioniSeyed Habibollah KaliliGian Luca GraziSeyyed Mojtaba MousaviGianandrea PasquinelliShah NazirGiancarlo CondelloShahab AlizadehGiandomenico AmicoShahabaldin RezaniaGianluca BorghiniShahrokh HatefiGianluca CarnevaleShaker Hassan Ali EI-SappaghGianluca M. TartagliaShan JiangGianna DipalmaShangting YouGianpaolo Di BonaShanmugam SumathiGiovanna IezziShaobin WangGiovanna Maria DimitriShaohua ChenGiovanna OrsiniShaokoon ChengGiovanni AngiulliShazid Md. SharkerGiovanni BrunoShengzhang WangGiovanni E. GiganteShepard HurwitzGiovanni GaleotoSheraz AslamGiovanni L. BrunoSherif RashadGiovanni NanoSherine F. ElsawaGiovanni PaolinoShigetaka ShimodairaGiovanni RalliShih-Feng ChouGiovanni SignoreShih-Hsin HoGirish PattappaShijun ZhongGiulia PaparellaShilpa BhandiGiuseppe CovielloShing-Hong LiuGiuseppe La RoccaShintaro SukegawaGiuseppe MartanoShiqiang GongGiuseppe MaulucciShiva AkbarzadehGiuseppe PortellaShivnarayan PatidarGiuseppina GiniShixiong ZhangGiuseppina MalcangiShizuka UchidaGiuseppina SandriSho TsukamotoGiustino OrlandoShotaro NishitsujiGiusy TornilloShrikanth GadadGladius LewisShuanhu ZhouGladys HoShuhei YoshidaGloria González-AseguinolazaShumaila Aman TasneemGobika ThiripuranatharShunqing TangGoncalo MarquesShuowei CaiGongbo LongShuqiang WangGoo-Rak KwonSigurður BrynjólfssonGoran KvaščevSikandar I. MullaGottfried LemperleSilva JoãoGrażyna BudrynSilvia Becerra-BayonaGuan ZeyiSilvia DanteGuangli LiSilvia De FranciaGueorgui Kostov GueorguievSilvia GiovanniniGuilherme B. LucasSilvia MartuGuilherme CaetanoSilvia OchoaGulsim KulsharovaSimon AnnaheimGundula Gesine Schulze-TanzilSimon LyttonGuo Dong GaoSimon SchoechlinGuo Liang GohSimon W. RabkinGustavo De Conti Teixeira CostaSimona CavaluGustavo Roberto CointrySimona CeccarelliHabib HamamSimona SalernoHabibollah HaronSimona ViolinoHai Long WangSingh RajenderHai WangSipho MdandaHaipeng LiuSmaoui SlimHaishu LinSmith Kashiram KhareHaisong LinSofia StraudiHaldun M. OzaktasSolmaz Maleki DizajHamed KarkhanehchiSomchai AmornyotinHamed NosratiSompop BencharitHan KemperSonja GamsjaegerHan NingSonja Herres-PawlisHana DrahovskáSoo Min KimHanan A. Hosni MahmoudSooyeon LeeHanieh KhaliliSophia N. SangiorgioHan-Jun KimSoren U. NielsenHannah DaileySounak SahuHans TroppSpiros ParamythiotisHans-Oliver RennekampffSpyridon KintziosHao WangSrecko StopicHaobo YuanSreekanth VedagopuramHarald BoehmSrikanthan RameshHarald M. StaussStanislav A. EvlashinHarikumar RajaguruStanislav M. RangelovHariprasath ManoharanStanisław MlekoHarmeen GorayaStavros PoulopoulosHarold P. EricksonStefan JunneHarry YuStefan RaithHartmut F. WitteStefan SeidelHashim Talib HashimStefan WegerHashimoto-Torii KazueStefan ZwingenbergerHasi Rani BaraiStefania MoscatoHassan MehboobStefania RaimondoHaw-Ming HuangStefano BellucciHayato OhshimaStefano BiricoltiHazrat AliStefano CirilloHéctor Capella-MonsonísStefano FranginiHéctor QuezadaStefano Marco Paolo RossiHee-Sung ChaeStefano MarianiHeikki KyröläinenStefano PaganoHelena FelgueirasStefano RossiHelena KellyStephan ReshkinHemant KumarStéphane ChabaudHermann GillyStephen InbrajHermis IatrouStephen LewisHerwig BachmannSteve A. AdeshinaHiroki YokotaSteve ElderHiromitsu KobayashiSteven Van DorenHiroshi HaraStevo PopovicHiroshi OzawaSubhas BarmanHiroshi TakemoriSubhasish SahaHiroyuki TsuchiyaSudhahar VaradarajanHong HuangSudhir Kumar PaidesettyHong YuSudi PatelHongbo WangSufang LiuHongyu RenSuleiman Y. YerimaHoria OprisSumate ChaiprapatHornyák IstvánSunah KimHorst Dieter LemkeSung-Cheol KohHosam O. ElansarySurachai KarnjanakomHossam Magdy BalahaSurendra ShuklaHossein EslamiSuresh ThanguduHossein Maleki-GhalehSusan M. BowyerHoward A. YoungSusan SharfsteinHsiang-Yin ChenSusana B. BravoHsin-Chien ChenSusanne GrässelHsueh-Wei ChangSushila MaharjanHuey-Chun HuangSusi BurgalassiHugo M. OliveiraSusumu MatsukumaHuibin ZouSutherland MaciverHui-Woog ChoeSvetlana S. EfimovaHung Huey-ShanSvetlana V. GuryanovaHung-Chuan PanSwarup RoyHung-Wei YangSwee Leong SingHung-Yin LinSwetha SunkarHuu-Tuan TranSyed Ahmad Chan BukhariHyeonji KimSyed Furqan QadriHyun-Chang LimSyed Khairul BasharHyunil LeeSylvie Le BorgneHyunwook ParkSylwia BalutaI. P. KuranovaSzabolcs BéniIan M. RogersSzczepan PiszczatowskiIan TerrySze Wan Samantha ShanIan Y. Z. LianSzidónia LefkovitsIbrahim DemirciTadeusz AmbrozyIbrahim KassisTae Hyun KangIgnacio Bosch RoigTae Keun YooIgnazio Gaspare VetranoTae-Hwan KimIgor Iuco Castro Da SilvaTae-Jin KimIgor JakovcevskiTaia Abd El-MageedIgor JurakTakaaki SenbonmatsuIgor M. PaivaTakahiko MorotomiIgor PrudovskyTakahiro KannoIgor ZubryckiTakashi HoshibaIjaz GulTakayuki KawatoIkuya MiyamotoTakayuki NakagomiIlaria ZanottiTakeharu TsugeIldiko PeterTakeshi FujinoIllyas Bin Md IsaTakeshi KikuchiIlya KlabukovTakeshi OmasaIlya Nifant’EvTakuro TobinaI-Ming ChuTal Luzzatto-KnaanIntan Syafinaz Mohamed Amin TawakkalTalib M. AlbyatiIoan PeteanTam PaulIoan TudosaTamako HataIoannis FourmousisTamas VargaIoannis G. AviziotisTamas ZakarIoannis KarabagiasTania PenchevaIonela Andreea NeacsuTânia S. MoraisIqram HussainTao FengIrena IlicTao WenIrena JekovaTao ZhangIrene Buj-CorralTar IldikóIreneusz SowaTarek MohamedIrfan KhanTarik RashidIrina HeidTariq AliIrina LarionovaTaro MatsumotoIrina M. Le-DeygenTaro UraseIrina MateiTatyana PashirovaIrina SavinaTeerasak DamrongrungruangIrma DumbryteTeleky Bernadette-EmokeIsabel Cláudia Masson Poiares BaptistaTelma QuintelaIsabel F. AmaralTeng-Chun YangIsabel LegazTeresa Gonçalves RibeiroIsabella ChiarottoTeresa RussoIsabelle HuynenTeresita Paz-MaldonadoI-Shiang TzengTereza Cristina Cardoso SilvaIsidoro Garcia GarciaTetsuya TakahashiIsmaeil GhasemiThaigarajan ParumasivamIsmael Fuente MerinoThais MilessiIsmail IsmailThais Suzane Milessi EstevesIsmail ÖçsoyThanapong ChaichanaIsmini E PapageorgiouThanasis TsanasItzhak OrionTheam Soon LimIuliana AproduTheresa AtkinsonIurii S. StafeevThi Tuong Vy PhanIvan A. YaremenkoThi-Huong NguyenIvan BrandslundThirupathi RavulaIvan Cruz-AcevesThomas BolandIvan ĆukThomas Jan HeyseIván Padrón GonzálezThomas MastIvana DrvenicaThomas ProftIvana KholováThomas S. MoodyIvana SamarzijaThorsten KirschIvaylo ChristovTi ZhouIvona BagoTian LiuIzabela FeckaTilahun Ayane DebeleIzabela Nawrot-HadzikTim RosenowIzabela ZglobickaTimothy HughesIzumi MashimaTimothy PaderaJ. JamariTina B. MckayJ. Michael SorrellTissiana BortolottoJacek HoffmanTiziana CiarambinoJacek KawaTiziana PietrangeloJacek RybkaTodd K. MoonJacek SmerekaTomasz KloskowskiJacek WąsikTomasz KockiJack MerrinTomasz WolnyJacob Lester JaremkoTommaso RossiJacobo Hernández-MontelongoTommaso TabanelliJacobo TrebolTomoyuki IwataJaecheul YuTong YeJagdish Suresh PatelToni LassilaJaime Gallardo-AlvaradoTorbjörn LedinJaime MorenoTorsten K. MalmJaime OrtegonToru ItagakiJakub SzabelskiToru TaguchiJames Chung Wai CheungToshiki MiyazakiJames D. BensonToshinobu KazuiJames DunnTouraj EhtezaziJames Kit-Hon TsoiTrina E. TalleiJamir Pitton RissardoTsun-Thai ChaiJan CebulaTuomas NäreojaJan ChrastinaTzu-Sen YangJan ZidekUberto BortolottiJana JanockovaUday JammalamadakaJanez TronteljUjjal Kumar BhawalJang Si HyongUmberto LuciaJanitha WanasundaraUmberto M. MusazziJanna ShainskyUsha KimJanosch HellerUte WoehlbierJaouhar FattahiUtoomporn SurayotJaroslaw KrzywanskiVahid RakhshanJasjit S. SuriVaidotas MarozasJasmine SinhaValérie MassardierJaved AhmadValeriu Marin ȘurlinJavid HussainValner BrusamarelloJay RamapuramVan Tu DuongJayanta Kumar PatraVarun JeotiJayoung KimVasia S. MavrogianniJean E. AaronVasif HasirchiJeanne Wilson-RawlsVasilia FasoulaJean-Paul DouliezVasiliki N. IkonomidouJeevithan ElangoVassilios PanoutsakopoulosJelena Kilić PamukovićVassilios SikavitsasJelena Nesovic OstojicVassiliy TsytsarevJelka GerśakVasudev BallalJen-Chang YangVeenu MangatJennifer A. FaralliVeerappan R. MuthukkaruppanJennifer FangVeronika HyskovaJennifer HulingVesna RastijaJennifer Woodell-MayVicente P. MartinsJen-Yuan (James) ChangVicente PerezJeremy D. P. BlandVickery Trinkaus-RandallJerilyn A. TimlinVictor AtuchinJérôme DuisitVictor MangasJérôme RossignolVictor UjorJerritta SelvarajVictoria SalemJerzy MalachowskiVijayalakshmi ShankarJerzy MosiewiczVijayan DharanendranJerzy WisniewskiVikas JaitakJesse JupiterViktor I. SevastianovJessica RamellaViktor VíglaskýJessica SchiaviViktoria ShcherbakovaJia HuaVinay SinghJiahao HuangVincent FontaineJian YuVincenza LaforgiaJianguang SuVincenzo AbbateJianguo SunVincenzo MatteiJianhua ZhangVincenzo VindigniJianqi WangVinicius Silva CastroJianwei ShuaiVinit RajJianxun DingVinod KumarJiaquan YuVinod KushvahaJia-Yang JuangVinod SureshJie GaoViorel CircuJie ZhangVitaliy YakovynaJijo LukoseVitaly LevashenkoJin YuanVittorio RampaJing HanVivian De WaardJing JiangVlad CiobotaruJing TianVladan LucicJing ZhaoVladimir KnyazJingen ZhuVolkan ArısanJingjing WuVukoman R. JokanovićJin-Jia HuWagner QuintilioJinny YeaahWahyu CaesarendraJin-Oh HahnWai Lun LoJinrong MaWalfre FrancoJinxiang XiWalter CabriJinzhou ZhuWangxiao HeJiong ZhouWanhe WangJisoo ShinWei ChenJoanna BrysWei ChengJoanna BrzeskaWei FeiJoanna DobrzańskaWei Long NgJoanna KazimierowiczWei ShaoJoanna MichalowskaWei-Biao LiaoJoanna MystkowskaWei-Chiang HongJoão Henrique Ghilardi LagoWei-Chun LinJoão Henrique LopesWeifeng LinJoão Paulo CarmoWeifeng PanJoão Paulo do Vale MadeiroWei-hsuan YuJoao Ramalho-SantosWeijun QinJoão SantosWeilue HeJoaquin BarberaWen Lin ChaiJoaquín Luis Sancho BruWen-Cheng ChenJochen StrubeWendong XiaoJohan KreugerWendy BollagJohannes Maximilian AlbesWendy Lynne GilleardJohn A. Albert St. CyrWenli ZhangJohn D. PerryWen-Pin HuJohn G. HardyWenqian ChenJohn G. PappasWenqiang GuJohn HulmeWerner BaumgartnerJohn MackayWidiastuti SetyaningsihJohn V. ForresterWilfried A. KuesJohnny PaduloWill K. ReevesJoias HammanWilliam H. FissellJon Einar DahlWilliam R. ReedJonas A. PramuditaWilliam ZimmermanJonas AlexandreWillie VannJonathan GrasmanWing-Hoi CheungJonathan R. DimmockWito RichterJonathan R. SilvaWojciech JelskiJonghoe ByunWojciech SitekJongsang SonWon-Kyu LeeJoo-Hyun SeoWoojin KangJorge Luís Victória BarbosaWoong Sub ByunJorge MoraisWu TingtingJorge N. R. MartinsWu WangJoris A. Van DongenWujie ZhangJosé A. MartinsWuyi MingJosé Angelo BarelaXavier PascalJosé Antonio Morales-GonzálezXi LouJosé António SimõesXianda ZhaoJosé Bañuls-RocaXiang LiJose Ignacio Priego QuesadaXiaodong ChenJosé Luis Pérez-CastrillónXiaodong LiJosé Luis Vázquez NogueraXiaohong WangJosé Luis Viveros-CeballosXiaohu XiaJose M. MuletXiaojian ShiJose Maria Pereira De GodoyXiaojie XuJose MerodioXiaojun WeiJosefa Isasi MarínXiao-Kun OuyangJosep Arnabat-DomínguezXiaoqing LvJoseph W. TurekXiaying XinJosé-Victor RodríguezXiming GuoJoshua JoshuaXin ChenJoy Iong-Zong ChenXin GaoJozsef KatonaXin GeJozsef VighXin LiJuan Bautista De SanctisXinchen WuJuan C. CruzXinda LiJuan Dominguez-RoblesXinfeng WeiJuan Ivan Nieto-HipolitoXingbang YangJuan Luis Pichardo-MolinaXinjie ZhangJuan Manuel Muriel-SánchezXinjun FengJuan Sepúlveda-AriasXinyan ZhangJude HemanthXisheng WengJuergen DreweXu ZhouJui-Chieh ChenXuan MeiJúlia Didier Pedrosa De AmorimXudong CaoJulio Calleja-GonzalezXuefei TanJumaa Salman ChiadXueming ZhangJun LuoXuewu LiJun SawaiXufeng XueJunaid AhmedXuming MaoJun-Gyu ParkYamini KrishnanJunhui PengYan PeiJu-Ock NamYan ZubavichusJürgen SchlegelYang CaoJürn W. P. SchmelzerYang HuJya-Wei ChengYang JiangK. KenryYang YuKah Hui WongYanhui GuoKai YuYannis KaramanosKaikai XuYaodong GuKalyanasundaram MadhuYaoxiang LiKalyani NairYasemin M. AkayKamal A. M. Abo-ElyousrYashdeep PhanseKamal Hany HusseinYasuhiko TabataKamalendra SinghYasuhiro KanataniKamil KaminskiYasuhiro TakahashiKamilla SwiechYasumasa OkazakiKang Hao CheongYasushi ShibataKang HuangYasutaka KitahamaKannan SridharanYasuyuki MoritaKanta TsumotoYaswanth KuthatiKaren LibyYatao RenKarin Bakran-LeblYawei DuKarina KwapiszewskaYean Ling PangKarolina ChilickaYehong ZhuoKarolina SzulcYenisel Plasencia CalañaKarthik RamamurthyYeoheung YunKatarina MihajlovskiYeon-Mo YangKatarína ValachováYeou-Jiunn ChenKatarzyna BułkowskaYi HongKatarzyna StaszakYi YangKatarzyna SzepietowskaYifan DaiKaterina StamatelatouYifan MaKathrin Bellmann-SickertYifeng CaoKatsura TakanoYih-Sharng ChenKavindra Kumar KesariYi-Hung ChiangKawsar AhmedYiming ZhangKazım KöseYing LuoKazuyuki TakaiYiqiang FanKe HuangYirong ChenKeiichi MatsubaraYiwen MengKeiji ShinozukaYiyao ChangKeith RochfortYiyu CaiKejun WuYiyuan HanKelvin Ian AfrashtehfarYong ChenKemal GörürYong YangKen LeungYongjian NianKen-ichi KimuraYongqin LiKenneth BaderYoshiaki AdachiKenneth R. FosterYoshifumi SaijoKenny Yat Hong KwanYoshiharu KimuraKensuke KumeYoshihide HashimotoKevin AndersonYoshihiro FukumotoKe-Vin ChangYoshihisa HirakawaKevin ItaYoshikazu WashizawaKhashayar KhoshmaneshYoshimi Enose-AkahataKhrystyna HarhayYoshito IkadaKimon DivarisYoshiya OdaKirill ZhichkinYounes MoussaouiKlaudia BanachYoung Yul KimKlaudija Carović-StankoYounghan YoonKlaus EmmanuelYounghoon ChoKoji OtaniYoung-Jae NamKonstantin Johannes ScholzYoussef W. NaguibKonstantin KozlovYu ZhangKonstantinos PetsiosYu ZhaoKourosh Kalantar-ZadehYu. E. GorshkovaKourosh ZareiniaYuan-Fong Chou ChauKrishan K. VermaYuanrong LuKrishna KantYu-Chao ChangKrystyna OlczykYuchen JiangKrzysztof BiernackiYu-Cheng ChouKrzysztof KurzydłowskiYudong ShenKrzysztof SzymkiewiczYu-Fang ShenKrzysztof TomczukYujie SuKuang-Tai KuoYukinori MaruoKulbhushan TikooYulia KiyanKumar GanesanYun Jung HeoKun LiangYuncheng DuKundan SivashanmuganYung-Kuo LeeKunihiko HiramatsuYung-Shun JuanKuo-Ching MeiYunsong LiuKuo-Kun TsengYunus AlapanKuo-Sheng ChengYupeng LüKwun Kei NgYusuf SertKyoung KimYusuf WibisonoKyung Hwan JungYusuke FujitaLaith AlzubaidiYutaka TamaruLakiesha WilliamsYuwei FanLakshmikanta MondalYuxin TongLamar O. MairYuxin ZhangLarisa RyskalinYves GourinatLars NorgrenZaal KokaiaLasse LindahlZahra DetakhshanLaszlo HejjelZaixing JiangLaura GastaldiZakariya AlgamalLaura Grațiela VicaşZar Chi SoeLaura GutiérrezZeeshan BandayLaura MangiaviniZenon LukaszewskiLaura R. JarboeZhaowei ChenLaurent DufosséZhaozhu ZhengLe YuZhen DingLei LiuZhen WangLei NieZheng ZhongLei WangZhengwei HuangLei ZhangZhicheng YaoLeo L. TsaiZhifeng JingLeonard AtanaseZhiguo HeLetao YangZhijie WangLetizia PenolazziZhiwei MenLia RimondiniZhiyuan HanLianyong QiZhizhu HeLibera FresielloZhong LiLichun BaiZhongchao ZhaoLilach SoreqZhuofu LiuLiliana ChemelloZhuojia FuLingwen DingZhuoran JiangLionel GamarraZhuoran MaLionel PichonZi-Bing JinLirong WangZihao OuLisa BiasettoZitong YuLiudmila Gerasimova-MeigalZixiang LiuLiudmila LeppikZizy Ibrahim ElbialyLoic LemonnierZoltán GáspáriLong LiuZoltan VerebLong QueZongdi FengLong-Sheng ChenZsuzsanna RecsanLorelei Irina BrasoveanuZulfiqarali G. AbbasLorenzo CostaZuzana KozovskaLorenzo Faggioni



